# The Prospect of Nanoparticle Systems for Modulating Immune Cell Polarization During Central Nervous System Infection

**DOI:** 10.3389/fimmu.2021.670931

**Published:** 2021-06-23

**Authors:** Lee E. Korshoj, Wen Shi, Bin Duan, Tammy Kielian

**Affiliations:** ^1^ Department of Pathology and Microbiology, University of Nebraska Medical Center, Omaha, NE, United States; ^2^ Mary & Dick Holland Regenerative Medicine Program, Division of Cardiology, Department of Internal Medicine, University of Nebraska Medical Center, Omaha, NE, United States

**Keywords:** central nervous system, infection, biofilm, immunometabolism, nanoparticles, blood-brain barrier, leukocytes, microglia

## Abstract

The blood-brain barrier (BBB) selectively restricts the entry of molecules from peripheral circulation into the central nervous system (CNS) parenchyma. Despite this protective barrier, bacteria and other pathogens can still invade the CNS, often as a consequence of immune deficiencies or complications following neurosurgical procedures. These infections are difficult to treat since many bacteria, such as *Staphylococcus aureus*, encode a repertoire of virulence factors, can acquire antibiotic resistance, and form biofilm. Additionally, pathogens can leverage virulence factor production to polarize host immune cells towards an anti-inflammatory phenotype, leading to chronic infection. The difficulty of pathogen clearance is magnified by the fact that antibiotics and other treatments cannot easily penetrate the BBB, which requires extended regimens to achieve therapeutic concentrations. Nanoparticle systems are rapidly emerging as a promising platform to treat a range of CNS disorders. Nanoparticles have several advantages, as they can be engineered to cross the BBB with specific functionality to increase cellular and molecular targeting, have controlled release of therapeutic agents, and superior bioavailability and circulation compared to traditional therapies. Within the CNS environment, therapeutic actions are not limited to directly targeting the pathogen, but can also be tailored to modulate immune cell activation to promote infection resolution. This perspective highlights the factors leading to infection persistence in the CNS and discusses how novel nanoparticle therapies can be engineered to provide enhanced treatment, specifically through modulation of immune cell polarization.

## Introduction

The blood-brain barrier (BBB) represents a double-edged sword in the context of central nervous system (CNS) infectious diseases. On the one hand, tight junctions between brain capillary endothelial cells, reinforced with astrocyte end feet and pericytes, act as a defense to restrict pathogen invasion into the CNS from the periphery (1, 2). However, the same tight junctions also hinder the delivery of therapeutics to the brain parenchyma in situations where the BBB is breached. A wide range of bacteria, viruses, fungi, and parasites can traverse the BBB with neurotropism for CNS meningeal, ventricular, and parenchymal compartments (1–3). These pathogens are responsible for severe clinical conditions including meningitis, encephalitis, and pyogenic infections. Patients with CNS infections often require lengthy hospitalization, critical care support, complex diagnostic tests, and invasive treatment procedures. Globally, more than 1.2 million individuals are affected by meningitis annually, with bacterial meningitis responsible for 120,000 deaths (4, 5). Many of the pathogens that invade the CNS are opportunistic and exploit patients with primary immune deficiencies that worsen disease severity (6, 7). Other CNS infections can arise from complications following neurosurgical procedures, such as craniotomy and cerebrospinal fluid (CSF) shunt placement (8–10). The expanded use of therapeutics targeting immune effector mechanisms, such as monoclonal antibodies to inhibit cytokine action or leukocyte trafficking, can increase susceptibility to CNS infection (11–13). In the CNS, pathogens can tightly regulate virulence factor and metabolite production to promote their survival (3, 14–16). In bacterial strains such as *Staphylococcus aureus*, this includes biofilm formation and antibiotic tolerance (17). Additionally, host-pathogen crosstalk can polarize immune cells towards an anti-inflammatory phenotype to promote chronic infection. Although CNS infections are generally less frequent compared to the periphery, their high morbidity and mortality rates necessitate better understanding and management to improve patient outcomes.

Treatments for CNS infection depend on the suspected pathogen, but one commonality exists – time is essential. As infections can be rapidly fatal, it is imperative that therapeutic interventions are initiated as soon as a diagnosis is made. For drugs, CNS entry is dependent on size, charge, lipophilicity, plasma protein binding, affinity for active transport mechanisms at the BBB, as well as edema and CSF flow (18). With these stringent requirements, it is no surprise that the BBB is the bottleneck of the pharmaceutical industry for CNS therapeutics. Around 98% of brain-targeting drug candidates have impeded ability to pass the BBB, including new classes of biotherapies such as RNAs (19, 20). Current treatment options for many bacterial, fungal, and viral pathogens are highly empirical due to a lack of clinical trial-based evidence and few approved therapies (3). Administration routes are also empirical, and due to the difficulty in achieving therapeutic concentrations of compounds in the CNS following intravenous injection, more invasive transcranial delivery is often required. This includes intrathecal and intraventricular injection of anti-infection agents dosed as high as 10-fold in excess of the minimum inhibitory concentration to achieve clearance, and ventricular catheters must be maintained for 24-48 h or substantially longer (21). A growing number of CNS infections with multi-drug resistant (MDR) bacteria such as *Acinetobacter baumannii*, *Pseudomonas aeruginosa*, and *Klebsiella pneumoniae* present a serious problem as these superbugs are only sensitive to select classes of polymyxin last-resort antibiotics, severely limiting treatment options (22). Further complicating treatment is that many drugs, such as the antibiotics for MDR bacteria, are associated with neurotoxicity due to the need for high therapeutic concentrations, non-specific targeting, and only small amounts of drug reaching the infection site within the CNS. As such, treatments must include neuroprotective agents to alleviate harmful side effects.

**Figure 1:**
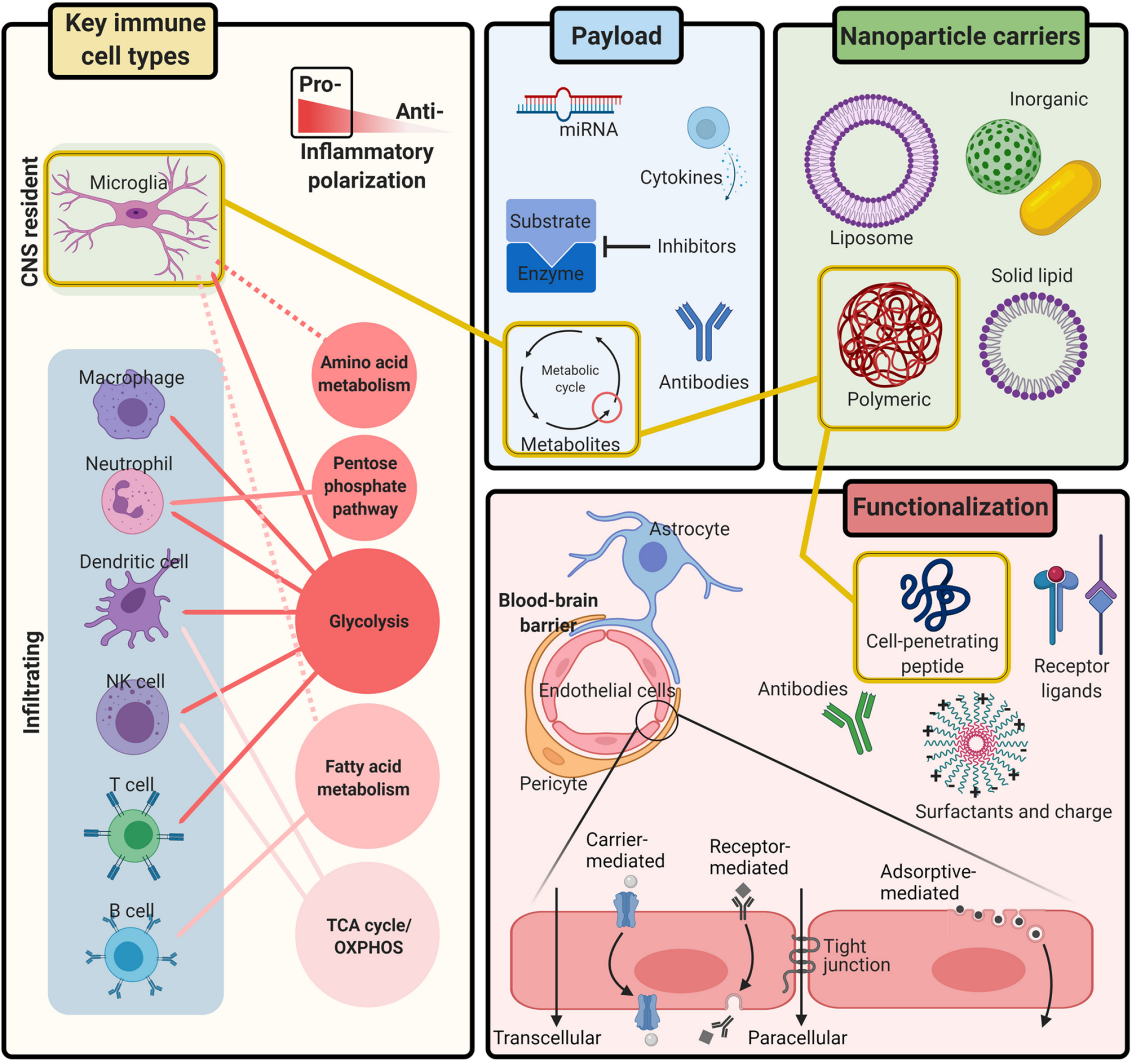
Integrating immunometabolism and nanoparticle systems for the treatment of CNS infection. Immune activation is controlled by the metabolic pathways needed to generate the energy and intermediates required for effector responses. Research continues to uncover the metabolic pathways that regulate inflammatory polarization of all **Key immune cell types** during CNS infection, including microglia and infiltrating leukocytes. **Nanoparticle carriers** can be engineered with different **Functionalization** to safely, and non-invasively transport therapeutic **Payloads** across the BBB to the CNS with a variety of tunable compositions, chemical ligands, and physiological characteristics. Together, nanoparticle systems provide a multi-tool kit of customizable parts for delivering immunometabolic modulating therapies to targeted cells in the CNS. Figure created with BioRender.

Engineered nanoparticle systems have emerged as a promising therapeutic path to circumvent BBB restrictions and provide targeted delivery of drugs to the CNS (23, 24). Additionally, the concept of using immunometabolic modulation to treat neurological disorders such as Alzheimer’s disease (AD), Parkinson’s disease (PD), and multiple sclerosis (MS) has gained traction in recent years (25, 26). We believe that using nanoparticle delivery systems with immunometabolic therapies could provide a paradigm shift for the successful treatment of life-threatening CNS infections. This approach has the potential as a dual-action therapeutic bolstering the host defenses and synergizing with anti-infection agents, ultimately improving patient outcomes (**Figure 1**).

## Pathogenic and Immune Characteristics of CNS Infections

### Mechanisms of Pathogen Entry Into the CNS

A variety of routes facilitate pathogen entry into the CNS (4). One common path is through the meninges and CSF. Bacterial species including *Streptococcus pneumoniae* and *Listeria monocytogenes* access the blood and CSF after colonization in the nasopharynx or gastrointestinal tract, respectively (27, 28). Once in the subarachnoid space, interactions between bacterial and host proteins facilitate invasion into the CNS parenchyma. For example, *S. pneumoniae* uses the adhesion molecule RrgA to bind the polymeric immunoglobulin receptor plgR or platelet-associated cell adhesion molecule (PECAM)-1 on endothelial cells (27). *L. monocytogenes* uses the internalin InlF to interact with the cytoplasmic intermediate filament protein vimentin that is also expressed on the surface of brain endothelial cells (28). Fungal invasion of the CNS can also occur through the CSF in cases of congenital, acquired, or drug-mediated T cell dysfunction (29, 30). Direct infection and replication inside BBB endothelial cells provides another route for pathogen entry to the CNS. For example, Zika virus is known to have tropism for vascular endothelial cells though mechanisms involving the AXL tyrosine kinase receptor family, and the protozoan *Toxoplasma gondii* utilizes parasite adhesion microneme protein-2 (MIC2) for growth in brain endothelial cells (31, 32). Upon replication, these pathogens are released into the CNS parenchyma after endothelial cell lysis. Microbes can also use host endocytic machinery to reach the CNS *via* transcytosis. For example, *S. pneumoniae* can cross endothelial barriers by clathrin- and caveolae-mediated micropinocytosis (33). West Nile virus (WNV) can invade the CNS through the use of lipid rafts and caveolae-facilitated endocytosis (34). Fungal species such as *Cryptococcus neoformans* also leverage host proteins for transcytosis, including cysteinyl leukotrienes and the glycoprotein receptor CD44 (35). Another notable entry route to the CNS for pathogens is *via* a “Trojan-horse” mechanism, whereby microbes are transported across the BBB within phagocytic leukocytes (36, 37). Research has demonstrated that WNV is carried to the brain *via* infected neutrophils, and CNS infection with *T. gondii* is associated with migration of infected monocytes and dendritic cells (DCs) (38, 39). Finally, foreign bodies introduced into the CNS provide direct routes for pathogen colonization, often leading to infection with skin flora such as *S. aureus* or *S. epidermidis* (40, 41). Later, we will discuss how the same biological mechanisms exploited by pathogens to enter the CNS can be used for designing new classes of nanoparticle therapeutics with enhanced BBB permeability.

### The Host Immune Response to CNS Infection

The immune response to pathogen invasion of the CNS is an organized and dynamic process. Microbes are sensed by microglia and astrocytes in the CNS parenchyma as well as macrophages within the choroid plexus, meninges, and perivascular space (42, 43). Activation occurs through the recognition of pathogen-associated molecular patterns (PAMPs) by a range of pattern recognition receptors (PRRs), the most well studied being the Toll-like receptor (TLR) family (44, 45). Microglial and CNS macrophage activation in response to TLR stimulation is characterized by increased major histocompatibility complex class II (MHCII) and costimulatory molecule (CD80 and CD86) expression. Additionally, pro-inflammatory cytokines and chemokines including TNF-α, IL-1β, CCL2, and CCL5 are secreted concomitant with nitric oxide and reactive oxygen species (ROS) production. Changes in phagocytosis, cell motility, and proliferation are also observed. These attributes serve to limit pathogen expansion, and recruit and activate peripheral blood leukocytes into the CNS to mitigate the infection. Ideally, activation is tightly regulated and short-lived before resolving into a homeostatic state characterized by the secretion of anti-inflammatory signals, including IL-10 and transforming growth factor-beta (TGF-β) that support neurorepair (44, 46, 47). Given the high mortality rates associated with CNS infections, it is clear that immune activation can become dysregulated, leading to bystander damage of surrounding normal brain parenchyma and increased disease severity.

In recent years, the rapidly expanding field of immunometabolism has demonstrated that immune activation is controlled by the metabolic pathways needed to generate the energy and intermediates required for effector responses (26, 48, 49). The major pathways identified to date that dictate leukocyte function include glycolysis, the tricarboxylic acid (TCA) cycle, oxidative phosphorylation (OXPHOS), fatty acid oxidation and synthesis (FAO and FAS, respectively), the pentose phosphate pathway (PPP), and amino acid metabolism (50). During normal resting conditions, leukocytes tend to display a basal activity of all major metabolic pathways. Glucose is converted to pyruvate to fuel the TCA cycle and generate adenosine triphosphate (ATP) for energy as well as nicotinamide adenine dinucleotide (NADH) and flavin adenine dinucleotide (FADH_2_) as electron donors for OXPHOS. Upon activation, cells undergo metabolic reprogramming characteristic of altered fuel consumption, modified mitochondrial structure and dynamics, preferential use of specific metabolic pathways, and metabolite flux (48–50). In response to pro-inflammatory signals, many leukocytes undergo Warburg metabolism that is typified by increased glycolysis under aerobic conditions (51). This glycolytic bias enhances the synthesis of nucleotides, amino acids, fatty acids, and other metabolic intermediates to promote proliferation and cytokine production, including rapid ATP generation. Cells in an anti-inflammatory state tend to favor OXPHOS since their biosynthetic demands are less pronounced. However, it is important to note that the concept of metabolic bias is not an “all-or-none” phenomenon but instead exists on a spectrum since metabolic pathways are highly integrated (50). Furthermore, unique metabolic pathways have been linked to specific cell types, revealing another layer of complexity (52–54). Metabolic programming is also highly dependent on substrate availability. This provides an opportunity for pathogens to manipulate host defenses through substrate competition that can ultimately suppress pro-inflammatory responses by biasing leukocytes towards an anti-inflammatory state (55, 56). For example, *S. aureus* biofilm promotes an anti-inflammatory milieu through depletion of key nutrients such as glucose, preferential recruitment of granulocytic-myeloid-derived suppressor cells (G-MDSCs), and release of lactate to drive production of the anti-inflammatory cytokine IL-10 (57). The CNS has a distinct nutrient environment compared to the periphery, which likely influences the immunometabolic status of resident microglia and infiltrating leukocytes during infection. While comprising only 2% of the total body mass, the brain utilizes approximately 25% of the glucose consumed by the human body (58). Under conditions of diminished glucose supply, such as infection or ischemia, CNS cells can adapt to use alternative energy sources generated from FAO or glutaminolysis (53). The concept of metabolically reprograming cells to promote infection clearance presents an exciting therapeutic opportunity. To realize this idea, it is important to understand the relationships between inflammatory polarization and metabolic status for the various immune cell populations within the CNS and how this changes in the context of infection.

### Immunometabolism of Glial and Leukocyte Populations

The key players in controlling CNS infections are resident microglia and macrophage populations along with infiltrating leukocytes. These cell types share many similarities in terms of TLR usage but also significant heterogeneity in effector functions. Microglia originate from the primitive yolk sac during development and comprise 5-10% of the total cell population in the brain parenchyma (59). During normal steady-state conditions, microglia survey the brain parenchyma detecting neuronal activity and maintain homeostasis through synaptic pruning, clearance of apoptotic cells, and regulating neurogenesis (60, 61). In response to pro-inflammatory stimuli, microglia undergo Warburg metabolism, shifting from OXPHOS in the resting state to aerobic glycolysis (62, 63). As a result, specific metabolite transporters and glycolytic genes are upregulated, notably the glucose transporter GLUT-1 and hexokinase, respectively, leading to protein acetylation due to acetyl-CoA accumulation and release of IL-1β. Further, superoxide generation is used to kill pathogens, and it is suggested that histone deacetylase (HDAC) activity links epigenetic changes with metabolism (25, 62). Non-immune cells, such as CNS resident astrocytes and oligodendrocytes also play key metabolic roles to support neuron homeostasis. Under physiological conditions, astrocytes provide neurons with metabolic substrates for neurotransmission, maintain neural electrical activity, and support energy balance and synaptic pruning (64, 65). Upon activation, astrocytes have been shown to undergo aerobic glycolysis to promote pro-inflammatory signals (54, 63). Oligodendrocytes form the lipid-rich myelin supporting the propagation of neuronal action potentials, where cells respond to glutamatergic signals by increasing glycolysis to support axonal energy metabolism (26, 66). The metabolic changes that occur in astrocytes and oligodendrocytes during CNS infection and how this shapes neuronal survival remain to be determined.

Infiltrating leukocytes are the other key contributors to CNS infection. Macrophages, neutrophils, DCs, and natural killer (NK) cells are rapidly recruited into the infected CNS where they can influence glial activation through release of inflammatory cytokines and other factors such as ROS (25, 26, 67–69). Macrophages and monocytes are found in the CNS meningeal and perivascular interfaces as well as the infected brain and experience a metabolic shift from OXPHOS to glycolysis upon pro-inflammatory activation, similar to microglia (70–72). The most comprehensive immunometabolic studies to date have been conducted on macrophages, wherein two major breakpoints in the TCA cycle result in succinate and citrate accumulation and nitric oxide, IL-6, and IL-1β production. Citrate accumulation also leads to the generation of itaconate, which exerts bactericidal activity (73). However, chronic production of itaconate can elicit anti-inflammatory effects and, as such, this balance must be tightly regulated. Similar to macrophages, pro-inflammatory DCs exhibit a metabolic shift towards glycolysis; however, DCs continue to use the TCA cycle for generating ATP as opposed to heavily relying on glycolysis which differs from macrophages (74, 75). There are numerous DC subsets, and it is important to recognize that each may undergo unique metabolic programs during activation in a context-dependent manner (76). Activated neutrophils favor glycolysis as well as the PPP to produce NADPH for redox reactions. Their low mitochondrial abundance reflects their reduced reliance on OXPHOS (77). NK cells do not experience a glycolytic bias upon activation but instead enhance both glycolysis and OXPHOS, where glucose remains the primary fuel (78). With regard to adaptive immunity, T cells also play important roles in many CNS infectious diseases, ranging from cytotoxic activity during viral infections to promoting innate immunity through the release of cytokines such as IFN-γ and IL-17 (79). Like their innate counterparts, T cell activation is highly dependent on glycolytic metabolism for their effector functions. However, metabolic variability exists for dictating T cell subset fate. For example, the OXPHOS pathway is important for Th17 differentiation, and the absence of OXPHOS during differentiation leads to regulatory T cell (Treg) development (79, 80). B cells are rather unique in their metabolic program compared to other immune cells, relying heavily on FAO and minimally on glycolysis (81, 82). There are few reports on the role of B cells during CNS infections, but available evidence shows important contributions for pathogen neutralization by enhanced opsonophagocytosis and complement activation (83). The metabolic diversity of infiltrating leukocytes during CNS infectious diseases and how this shapes not only their intrinsic properties but also extrinsic effects on surrounding leukocytes and resident glia represents a complex scenario, and one that is ripe for interrogation to exploit pathways that promote infection resolution without excessive bystander damage to normal brain parenchyma.

### Modulating Immune Cell Polarization

Extensive evidence has shown that immune cell polarization is linked to metabolism, supporting the idea of manipulating metabolism as a means to direct immune cells towards pathways that promote infection clearance, which has been coined metabolic reprogramming (50). Most current research into immune modulation in the CNS has targeted inflammation associated with AD, PD, and MS; however, the same concepts can be leveraged for CNS infectious diseases. In the context of neurodegenerative disorders, T cell activation has been targeted to attenuate chronic inflammation. Initial work showed that inhibition of glycolysis limited T cell pathogenicity by favoring Treg development (84, 85). Tetramerization of pyruvate kinase M2, the enzyme catalyzing the last step in glycolysis, inhibited the glycolytic activity of pro-inflammatory T cells to ameliorate experimental autoimmune encephalomyelitis (EAE), the mouse model of MS (86). Other work demonstrated that the TCA derivative itaconate also reduced EAE severity by suppressing T cell and microglial activation (87). Further studies have shown metabolic polarization effects in T cells with cytokines such as IFN-β and targeting mitochondrial respiratory chain enzymes (88, 89). A growing body of literature is beginning to uncover the mechanisms driving microglial plasticity in the brain, where the mechanistic target of rapamycin (mTOR) pathway has been identified and has clear links with metabolism (90). As critical metabolic nodes emerge, a variety of approaches relying on pharmacological agents, cytokines, lipid messengers, and microRNAs have all been shown to be effective metabolic modulating agents (91).

Insights into how metabolic status may shape CNS immune activation can also be drawn from research in the periphery, where much focus has been on macrophages. Studies have uncovered mechanisms behind mitochondrial repurposing during activation, and how resulting mitochondrial reactive oxygen species (mtROS) production can be blocked to promote anti-inflammatory states (92). Other work has demonstrated that metabolic reprogramming of monocytes *via* the OXPHOS inhibitor oligomycin reduced bacterial burden in a *S. aureus* biofilm model of prosthetic joint infection (93). The effectiveness of this treatment resulted from inhibiting the anti-inflammatory OXPHOS bias, shifting cells towards a pro-inflammatory glycolytic state to promote biofilm clearance. Pertinent to CNS infection, similar immune-based approaches have been used with exogenous application of IL-1β or grafted pro-inflammatory macrophages, both of which lowered bacterial burden in a *S. aureus* biofilm model of craniotomy infection (94, 95). As another layer of complexity, a recent study demonstrated the influence of microenvironment in shaping immunomodulatory attributes, where macrophage expression of glycolytic markers was suppressed upon migration into the brain parenchyma (71). More specifically, lactate dehydrogenase A (LDHA; converts pyruvate to lactate) and monocarboxylate transporter 4 (MCT-4; exports lactate from glycolytic cells) expression was significantly reduced in macrophages that invaded the brain parenchyma in EAE, whereas these molecules were elevated in macrophages associated with perivascular cuffs. This suggests a failure of macrophages to maintain their pro-inflammatory properties upon entering the CNS, which the authors attributed to differences in metabolic demand. While a specific mechanism for this reprogramming is unknown, it could be influenced by local nutrient or metabolite availability, such as lactate itself, which is known to be produced by astrocytes and oligodendrocytes for supporting proper axonal function (58, 96), or it may provide balance to the local inflammatory response. Collectively, these findings support the idea that immune cell function could be tailored by modulating metabolism to overcome deficiencies in CNS metabolites, such that infiltrating leukocytes remain in a pro-inflammatory state to fight infection.

The aforementioned examples reflect only a small amount of the growing literature on metabolic modulation. Ongoing work continues to identify molecular agents targeting aspects of key metabolic pathways. Overall, strong evidence supports the use of metabolic modulation therapy for controlling immune cell activation states and effector functions (84–86, 89, 91, 94). The heterogeneity between different cell types highlights the need to uniquely target select immune populations. Additionally, more work should aim to investigate how immunometabolic therapies can synergize with existing anti-infection drugs to enhance clearance from the CNS. Such an immunometabolic approach to treating CNS infections has potential to improve disease outcomes, depending on the availability of suitable delivery mechanisms.

## The Prospect of Nanoparticle Systems for Modulating Immune Cell Polarization

### Shortcomings of Current CNS Infection Treatments

As previously discussed, the BBB is a cooperative interaction between brain capillary endothelial cells, astrocytes, and pericytes that maintains brain homeostasis and controls nutrient influx into the parenchyma. Transport through the BBB can occur through a variety of routes, generally classified as passive transport, carrier-mediated, and vesicular trafficking (**Figure 1**) (97). Passive transport is mostly limited to small substances. Small hydrophilic compounds may pass paracellularly through the tight junctions between endothelial cells likely by means of transient relaxation of the junctions, while small lipophilic substances can use transcellular passive diffusion to reach the brain (97). Carrier-mediated transport exploits diverse solute transporters for traversing the BBB, such as those for glucose or amino acids. Receptor-mediated and adsorptive-mediated transport utilize antibody binding or plasma proteins for crossing *via* endocytosis and pinocytosis (98).

Expectedly, delivery of anti-infection agents to the CNS is strongly hindered by the BBB, and more invasive transcranial delivery *via* intrathecal and intraventricular injection is often used as a bypass (99, 100). However, bypass strategies are complicated by limited drug diffusion, which reduces biodistribution to the target location in the parenchyma. Osmotic disruption of the BBB with vasoactive substances, exposure to high intensity focused ultrasound, and electromagnetic pulses have also been explored to improve drug permeability to the CNS (101–103). However, BBB disruption can lead to unwanted entry of other molecules into the CNS or drugs becoming trapped in brain endothelial cells rather than distributing to target sites. Engineered nanoparticles represent a promising approach to improve non-invasive delivery of CNS therapeutics by ferrying drugs across the BBB. Nanoparticles can be designed to perform multiple, targeted functions aimed at both the pathogen and host, and their biodegradable properties have the added advantage of self-clearance (20, 23, 24, 100).

### Design Variables of Nanoparticle Therapies

Nanoparticles are small structures ranging from 1 to 1000 nm in diameter. They can be generated by a wide array of biodegradable and non-biodegradable substances and readily modified to deliver therapeutic agents, as discussed in the following sections (24). There are several approaches for transporting nanoparticles across the BBB, all facilitated by harnessing the physiological properties of endogenous molecules required for proper brain function (98, 104). For example, carrier-mediated transport allows nanoparticles to use essential nutrient transporters, such as GLUT-1 for glucose and L1 and y+ for large amino acids. Through adsorptive-mediated transcytosis, electrostatic interactions between cationic ligands and negatively charged endothelial cell membranes lead to vesicle-based endocytosis. Perhaps the most effective approach, receptor-mediated transcytosis, relies on luminal plasma membrane receptors of endothelial cells for endocytosis. Examples include the lactoferrin and transferrin receptors (LfR and TfR, respectively), low density lipoprotein receptor-related protein 1 and 2 (LRP-1 and -2), insulin receptor, and folate receptor (98, 104).

To exploit the endogenous transport machinery of the BBB, nanoparticles must be designed to mimic physiologically active compounds. Several key characteristics can be leveraged to optimize nanoparticle entry into the CNS. First, nanoparticle size is crucial for endocytosis, with a critical limit of approximately 200 nm or less for efficient cellular uptake *via* clathrin-mediated endocytosis (23, 105). Charge is another important factor affecting both internalization and circulation time. Due to the net negative charge on endothelial cell membranes, positively charged nanoparticles can more readily use adsorptive transcytosis. On the contrary, neutral and negatively charged nanoparticles remain in circulation longer because of reduced protein adsorption. Zwitterionic nanoparticles can provide a balance between uptake and circulation requirements (106). Functionalization through incorporation of surface ligands provides the most flexibility to engineered nanoparticles. The main objective in selecting surface ligands is increasing BBB passage and cell-specific targeting through carrier- and receptor-mediated transcytosis. Studies have demonstrated the ability to decorate particles with ligands for GLUT-1, albumin transporters, LfRs and TfRs, and more (107–110). The use of cell-penetrating peptides as surface ligands can be used to bypass endocytosis, leading to direct nanoparticle entry to the cytoplasm (111). Studies have also demonstrated the use of ligands such as insulin for targeting affected brain regions in neurodegenerative and neuropsychiatric disorders (105). Not only the ligand itself, but its density or avidity are also important factors, as too many high affinity ligands can hinder endocytosis by anchoring nanoparticles to cell membranes (110).

Intravenous injection is the most widely utilized route for nanoparticle administration. However, the rapid clearance of particles from circulation can limit the concentrations reaching the CNS (23). New non-invasive routes of administration are being explored to improve CNS bioavailability. Intranasal delivery is a major alternative route, which could facilitate direct nose-to-brain delivery in a matter of minutes *via* olfactory and trigeminal nerves (112–117). The functional diversity and customization possibilities in designing CNS-targeting nanoparticles makes them multi-tool kits with options for tailoring transport routes, targets, and payload release kinetics. Researchers continue to discern the relative importance of the variables governing nanoparticle characteristics and how one property may modify another attribute (118). One such study examining these relationships determined that for the specific polymeric nanoparticles used, the most influential parameter for efficient BBB penetration was the surfactant type, whereas size and zeta potential had little impact (119). Continued efforts advancing CNS-targeting nanoparticles will only enhance their potential for personalized medicine applications.

### Nanoparticles for the CNS

Significant work has identified a wide range of polymeric, lipid-based, cell-derived, and inorganic nanoparticles as viable therapeutic options to promote CNS uptake. While most of the current research and select examples discussed below have focused on cancer, neuroinflammation, and neurodegenerative diseases, the same nanoparticle systems can be leveraged to treat CNS infections by simply changing the therapeutic payload. Both *in vitro* and *in vivo* studies have been conducted to demonstrate the vast potential of nanoparticle therapeutics. While *in vitro* systems are useful for isolating specific research variables and uncovering transport mechanisms, the use of *in vivo* models provides much greater measures of physiological relevance (97). The fact that a majority of the examples described below are from *in vivo* models shows the exciting success of many nanoparticle systems and the impending progression toward clinical trials.

Polymers, both artificially- and naturally-derived, have received the most attention for CNS delivery (24, 100, 120). The most widely used polymer is poly(D,L-lactide-co-glycolic acid) (PLGA), which is FDA approved and can undergo hydrolysis within the body to form biocompatible metabolites (121). PLGA nanoparticles have proven effective at increasing the half-life and stability of drugs such as the chemotherapeutic agent cisplatin, in comparison to the raw drug counterpart (122). Another study demonstrated that PLGA encapsulation of the anti-inflammatory and anti-oxidant compound curcumin dramatically improved BBB permeability and stimulated hippocampal neurogenesis to reduce cognitive decline in a rat model of AD (123). PLGA can also be conjugated and functionalized for specific targeting. In one example, researchers used Lf-conjugated polyethylene glycol (PEG)-PLGA nanoparticles containing the peptide urocortin to increase blood circulation time and promote specific uptake in the striatum and substantia nigra as a neuroprotective therapeutic for PD (124, 125).

Poly(alkyl cyanoacrylate) (PACA) is another nanoparticle polymer with proven ability to cross the BBB. PACA nanoparticles can be coated with surfactants for improved BBB permeability and have demonstrated promise as potential AD therapeutics from *in vitro* studies showing limited effects on vascular homeostasis and inflammatory response (126). Poly(butyl cyanoacrylate) (PBCA) nanoparticles are closely related to PACA, but degrade more rapidly in the body due to their higher water solubility (127). Other classes of biocompatible polymers include copolymer-poly(methylmethacrylate-sulfopropylmethacrylate) (PMMA-SPM), which have been loaded with anti-retroviral drugs for transport across the BBB (128). Natural polymers such as chitosan have also been explored as nanoparticle materials with CNS permeability. Tripolyphosphate cross-linked chitosan nanoparticles delivered the anti-inflammatory compound piperine to the CNS following intranasal administration in a rat model of sporadic dementia, which reduced inflammation by decreasing TNF-α and activated caspase-3 concomitant with increased superoxide dismutase activity (129). Another study used chitosan-coated lipid nanoparticle carriers conjugated to the transactivator of transcription (TAT) cell-penetrating peptide to enhance CNS delivery of glial cell-derived neurotrophic factor (GDNF) in a mouse model of PD, leading to decreased dopaminergic neuron loss and improved motor function (130).

Lipid-based nanoparticles include solid lipid and nanoemulsions, both of which are biocompatible, stable, and BBB-permeable (131, 132). Solid lipid nanoparticles consist of glycerides, waxes, and fatty acids stabilized with emulsifiers, and nanoemulsions are similar but with a liquid lipid core. Both are best suited for carrying lipophilic and hydrophobic drugs. A recent study used solid lipid nanoparticles loaded with doxorubicin for treating glioblastoma, which demonstrated excellent tumor cell toxicity (131).

Cell-derived nanoparticles consist of liposomes and exosomes. Liposomes have an aqueous core surrounded by a phospholipid bilayer, making them suitable for both hydrophobic and hydrophilic drugs. Phase III clinical trials are underway using cytarabine-carrying liposomes for treatment of neoplastic meningitis. The liposomal nanoparticles showed increased therapeutic concentrations of cytarabine in the CSF for up to 14 days post-administration (133). Another study has used cationic nanoliposomes with TfR-affinity ligands to deliver oligonucleotides and siRNA to the brain within 6 hours following intravenous injection. These nanoparticles reduced neuroinflammation when the siRNA targeted TNF-α (132). Exosomes are small vesicles secreted from all cell types that contain a wide range of biological molecules, including surface proteins, ligands, cytokines, and RNAs. They are beginning to be studied for therapeutic applications based on their ability to be loaded with drugs, BBB permeability, and potential for nasal administration (134).

Other unique nanoparticle formulations continue to be developed (135, 136). For example, biodegradable anti-TfR monoclonal antibody (OX26)-PEGylated selenium nanoparticles were shown to suppress pathological inflammation and oxidative metabolism associated with cerebral stroke (137). Additionally, inorganic gold nanoparticles with varying surface ligands have shown promise for treating CNS bacterial infections due to both the inherent bactericidal properties of gold and conjugated antibiotics (138). The nanoparticle examples noted here merely represent a small snapshot of the wealth of possibilities for designing therapeutic carriers for improved treatment of CNS infections.

### Cell-Specific Targeting With Nanoparticles

A final goal of nanoparticle therapies is cell-specific targeting (139, 140). In the context of CNS parenchymal infection, microglia represent a logical candidate. For microglial specificity, nanoparticles can leverage receptor-targeting ligands and the inherent phagocytic properties of microglia, while maintaining biocompatibility (140). An early study of microglial targeting used liposomal nanoparticles modified with the TLR4 ligand lipopolysaccharide (LPS), which significantly increased uptake of the encapsulated drug compared to non-targeted liposomes (141). In a later study, ceria-zirconia nanoparticles decorated with CD11b antibody showed preferential uptake by microglia compared to other cell types in the brain and higher internalization compared to nanoparticles conjugated to an isotype-matched control antibody (142). Other promising surface receptors exist to target microglia, including triggering receptor expressed on myeloid cells 2 (TREM2), Tmem119, and P2RY12 (143). Recent work has highlighted the significant transcriptional heterogeneity of leukocyte subpopulations within the CNS during *S. aureus* craniotomy infection, including microglia (144). The tunability of nanoparticle systems has exciting potential to target this diversity within a given cell type, where typical molecular therapies fall short. Of note, several of the receptors that have been exploited to deliver nanoparticles to microglia are also expressed on macrophages and neutrophils that infiltrate the CNS during infection. Therefore, targeting a single cell type with these receptors is unlikely. However, with the increasing abundance of next-generation sequencing datasets for CNS diseases, including infection, the identification of receptors that are enriched on a given phagocyte population is likely. Ultimately, nanoparticles targeting all of the key immune cell populations would fully complement the multi-tool kit of carriers for precisely modulating metabolic activity for the treatment of CNS infections.

## Discussion

Many bacteria, viruses, fungi, and parasites can invade the CNS and cause severe meningitis, encephalitis, and pyogenic infections. These conditions can become exceedingly dangerous as pathogens can acquire drug resistance, form biofilm, and leverage virulence factors that disrupt the host immune response and reprogram immune cells towards an anti-inflammatory bias. These challenges are exacerbated by the fact that therapeutic agent delivery to the CNS is hindered by the BBB, the same defense meant to exclude harmful pathogens. As such, treatment of CNS infections remains highly empirical and difficult, relying on extended and/or invasive delivery of anti-infection agents often with deleterious side effects.

We propose that together, the fields of immunometabolism and nanotechnology have the potential for a paradigm shift in novel treatments for CNS infections (**Figure 1**). The rapidly expanding field of immunometabolism has demonstrated that immune activation is controlled by the metabolic pathways needed to generate the energy and intermediates required for effector responses. The metabolic pathways that elicit pro-inflammatory activity have been described for all the key immune players in CNS infection, including microglia and infiltrating leukocytes but primarily in the context of neurodegeneration. It remains to be determined whether similar metabolic programs are observed during infection, which may differ based on nutrient competition with the pathogen. A variety of pharmacological agents, cytokines, lipid messengers, and microRNAs have been shown to modulate metabolism and could serve as potential therapeutics. In the realm of nanotechnology, nanoparticles can be engineered with a host of tunable structures, chemical ligands, and physiological characteristics to safely, and non-invasively deliver therapeutics to the CNS by transporting drugs across the BBB. Nanoparticle applications and design will continue to improve with increased knowledge of the precise interactions between structure, BBB penetration, and efficacy. Overall, merging therapeutic approaches with metabolic modulating agents and nanoparticles as delivery vehicles warrants the need for more focused research efforts given the promise for improving patient outcomes associated with CNS infections.

Research into metabolic reprogramming in the CNS to date has mainly focused on AD, PD, and MS, but more emphasis should be placed on infectious diseases, particularly in the current era of increasing antimicrobial resistance. Compared to peripheral tissues, the use of nanoparticles is especially important for CNS infections because of the BBB exclusivity. In the periphery, the major objective of nanoparticle usage is to target specific cell types and enhance cellular uptake of the drug or payload. In the CNS, these same attributes hold with the additional requirement of BBB penetration, which adds complexity to any potential therapeutic application. Nanoparticle-mediated metabolic modulation therapy could bolster endogenous cellular effector mechanisms to better fight infections compared to the introduction of compounds with harmful side effects throughout the CNS and periphery. Alongside future work into nanoparticle-based treatments for CNS infections, we anticipate the need for more long-term studies to address potential nanoparticle toxicity. Finally, we predict that the most effective nanoparticle therapeutics for CNS infections will be realized in a combinational platform leveraging not only metabolic modulation but also nanoparticle-encapsulated or intravenous anti-infection agents. The optimal metabolic modulation therapy may also not take the form of a single re-polarization event, but instead a series of controlled toggling between pro- and anti-inflammatory states to adjust to the temporal nature of inflammation as the infection subsides.

## Author Contributions

LK drafted the manuscript. WS, BD, and TK reviewed and revised the manuscript. All authors contributed to the article and approved the submitted version.

## Funding

The Kielian laboratory is supported by NIH grants R01 NS107369 and 3P01AI083211 (Project 4 to TK) and a Nebraska Research Institute Collaborative Grant (to TK and BD).

## Conflict of Interest

The authors declare that the research was conducted in the absence of any commercial or financial relationships that could be construed as a potential conflict of interest.
